# High pressure systems as sustainable extraction and pre-treatment technologies for a holistic corn stover biorefinery

**DOI:** 10.1186/s13065-021-00762-1

**Published:** 2021-05-29

**Authors:** Pakin Noppawan, Adrienne Gallant Lanctôt, Maria Magro, Pablo Gil Navarro, Nontipa Supanchaiyamat, Thomas M. Attard, Andrew J. Hunt

**Affiliations:** 1 Materials Chemistry Research Center, Department of Chemistry, Faculty of Science, Khon Kaen University, Khon Kaen, 40002 Thailand; 2 Green Chemistry Centre of Excellence, Department of Chemistry, University of York, Heslington, York, YO10 5DD UK; 3 RX Extraction Ltd., Unit 10, Rowen Trade Estate, Neville Road, Bradford, BD4 8TQ UK

**Keywords:** Corn stover, Green chemistry, Supercritical, Carbon dioxide, Water

## Abstract

This mini-review assesses supercritical carbon dioxide (scCO_2_) extraction and high-pressure carbon dioxide pre-treatment technologies for valorisation of corn stover agricultural residues with particular focus on showing how these can aid in the creation of a holistic biorefineries. Corn stover is currently the largest source of agriculture residues in the USA, as such there is significant potential for exploitation to yield valuable chemicals. ScCO_2_ extraction could lead to the recovery of a variety of different chemicals which include flavonoids, sterols, steroid ketones, hydrocarbons, saturated fatty acids, unsaturated fatty acids, fatty alcohols, phenolics and triterpenoids. Importantly, recent studies have not only demonstrated that supercritical extraction can be utilized for the recovery of plant lipids for use in consumer products, including nutraceuticals and personal care, but the processing of treated biomass can lead to enhanced yields and recovery of other products from biorefinery processes. Despite the great potential and opportunities for using scCO_2_ and high-pressure systems in a biorefinery context their real-world application faces significant challenges to overcome before it is widely applied. Such challenges have also been discussed in the context of this mini-review.

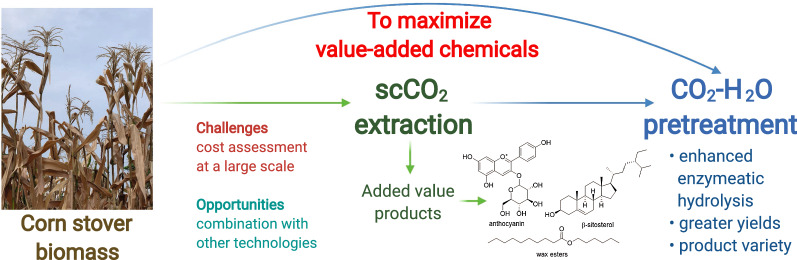

## Background

Increasing environmental issues, coupled with rising oil prices, have strengthened the interest in renewable feedstock use for both fuels and chemicals [1]. The biorefinery is an effective strategy to encourage the emerging future bioeconomy by offering a range of products from a broad spectrum of biomass sources, thereby fulfilling the various needs of society [2]. The concept of a biorefinery enables the valorisation of renewable feedstocks through maximum use of biomass in a holistic manner and thus generating minimal amounts of waste. Biorefineries can create opportunities for the development of new and more sustainable processes, leading to the generation of a wide range of products including chemicals, fuels, and materials [2]. The advanced biofuel production processes or secondary generation biorefineries can effectively exploit agricultural residues and non-food crops, hence making them a sustainable option for a bio-based chemical industry [3]. These feedstocks, such as corn (maize) stover, are often lignocellulosic, consisting of cellulose, hemicellulose and lignin that form a complex network. Comparing with other crop residues such as wheat straw, whose productivity is strongly influenced by fertiliser application during cultivation, corn stover biorefinery showed lower ecological impact and potential toxicity to humans [4]. Although the primary metabolites of corn stover are polysaccharides and lignin, important secondary metabolites such as waxes, terpenoids, and phenolic compounds such as lignans, account for up to 10% of the dry weight [5]. Research has focused on obtaining the maximum value from lignocellulosic biomass by developing holistic integrated processes in which a range of product streams are produced from one feedstock [1].

There has been growing interest in utilizing C4 biomass in a biorefinery. C4 plants, such as corn (*Zea mays ssp.*), have a higher photosynthetic efficiency than C3 plants leading to higher biomass production while at the same time utilize less water and nutrients [6, 7]. Corn stover, is an agricultural waste and refers to the residue left behind once the corn grain is harvested. It consists of the stalk, leaf, cob and husk tissues and it is estimated that it constitutes 27.2% of global agricultural residues [8].

Agricultural residues are typically left on the fields after harvests, are used as fodder for livestock, are disposed of in landfill or burnt in the field [9]. Corn stover is classified as a lignocellulosic biomass source, i.e. it is composed of the three major components cellulose, hemicellulose and lignin [10]. Currently, 5% is taken from the field for use as cattle feed and bedding. The rest is left to rot in the field to preserve soil carbon levels and prevent erosion [8]. As such, there is presently an appreciable amount of available corn agricultural residue. Significant attention has been given to corn stover as a promising primary feedstock selection for biorefineries [11]. The use of corn stover will not only aid in meeting the renewable energy goals but also provide another source of income for corn farmers [11]. In the US alone, stover has been reported as being the largest source of agriculture residues, with 17–77 million ton (under normal agricultural practices) and 64–139 million dry metric tons/year (under more extensive agricultural practices) of potentially available biomass for utilization as a sustainable feedstock. These figures are projected to increase to 56–127 (under normal agricultural practices) and 200–245 million dry tons per year in 2030 (under more extensive agricultural practices) [12, 13]. Furthermore, this quantity is likely to increase as the density at which the crops are being planted is increasing by 1000 plants/hectare per year [14].

**Fig. 1:**
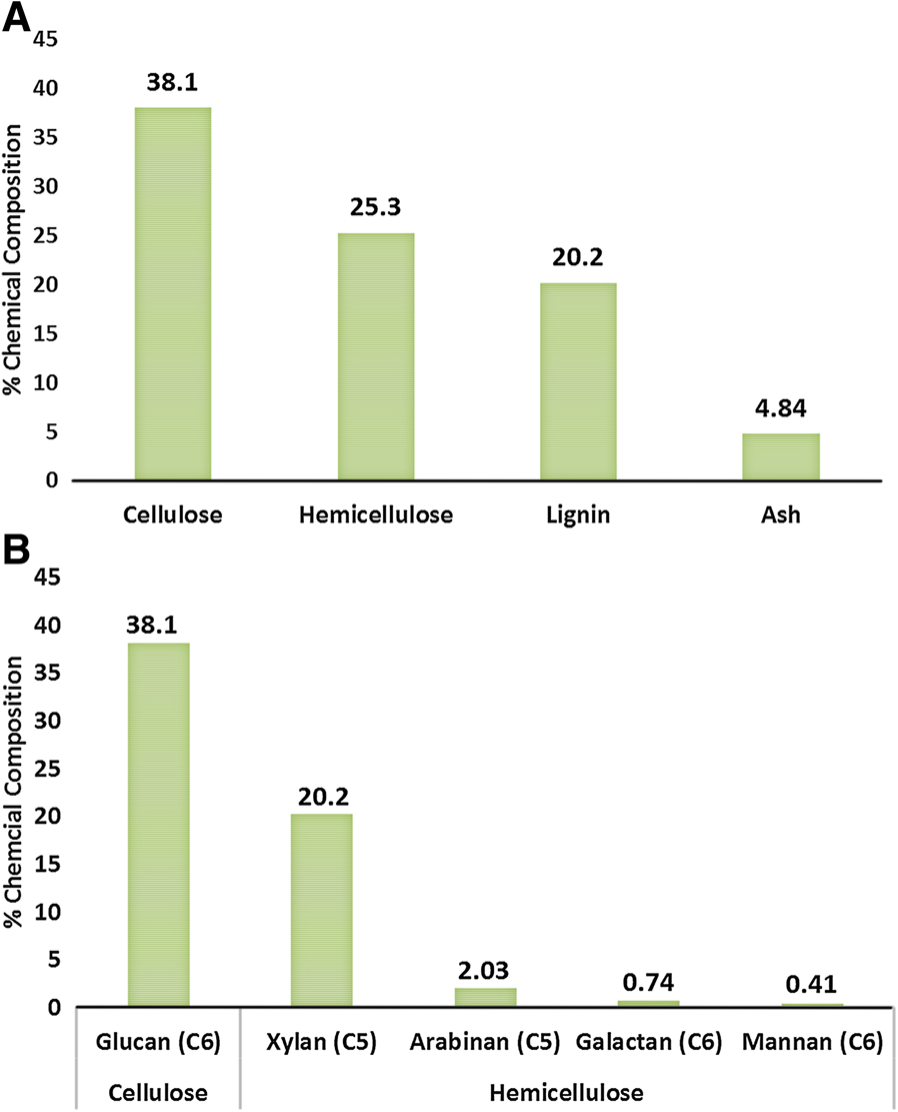
Composition of corn stover (**A**) % of major bio-polymers and ash present in stover, **B** sugars composition present in stover

The chemical composition (% cellulose, hemicellulose, and lignin) of corn stover used as raw materials for a biorefinery may be found in Fig. 1. The hydrolysis of the cellulose and hemicellulose gives rise to sugars that can be fermented to produce bioethanol. The lignin is high in phenolics and may be utilized in applications such as energy generation and chemical extraction. The relatively high ash content could be utilized for applications including the recovery of metals or the preparation of separation (chromatographic) materials.

To maximize the value of biomass, a holistic approach to feedstock use is required. This implies that every chemical component of the biomass must be exploited to generate a range of chemicals, to move towards a greener economy. Wang and co-workers recently reported the more holistic and efficient utilization of corn stalk by using supercritical water in the presence of oxidant for enhancement gasification of the by-product of cornstalk, depolymerization slag [15]. Several steps are needed for the complete processing of lignocellulosic biomasses such as corn stover. Extraction and pre-treatment of biomass are some of these steps where several green technologies can be utilized to obtain the desired products. Recent work has demonstrated that high pressure carbon dioxide systems could be commercially useful in the extraction and pre-treatment of biomass [16].

Extraction of natural products should include the removal of valuable metabolites or active compounds without any degradation of the biomass. This step is particularly relevant when the raw material is rich in high value compounds. Extraction has traditionally been performed using solvents and conventional heating methodologies. The limitations of these traditional methods include the use of large volumes of non-renewable and often toxic solvents (leading to problems in solvent removal and waste disposal), a greater flammability risk, relatively large extraction times required as well as the high temperatures required to heat the solvent to its boiling point. The latter could lead to modification of the extracted compounds as a result of thermal decomposition, hence reducing the quality of the extract [17]. Supercritical carbon dioxide (scCO_2_) extraction is an attractive alternative to traditional solvent extraction, as it has tuneable solvent properties, high diffusivity and is easily recycled. In addition, supercritical carbon dioxide extraction is not yet widely use in a corn stover biorefinery leading to the loss of valued molecules, thus scCO_2_ could be utilize as a first stage, prior to aggressive physical pre-treatment [18].

Pre-treatment is typically the disruption of the intricate lignocellulosic structure to facilitate downstream processing. Pre-treatment is considered as one of the most challenging steps in a biorefineries, since it can be the most expensive [5]. Furthermore, subsequent steps can be highly affected by the chosen pre-treatment method. There are several barriers that limit the hydrolysis of biomass including the crystalline structure of cellulose, the hemicellulose surrounding the cellulose, and the protective sheath of lignin; the latter of which binds the cells together reducing the total surface area for enzyme hydrolysis [19]. Pre-treatment can be divided into four main types: physical, chemical, physico-chemical, and biological. However, some of these methods can be combined to pre-treat certain biomasses, including corn stover. Many of these common methods use harsh conditions and chemicals, as well as requiring large amounts of energy. Furthermore, such conditions could degrade sugars forming furans and carboxylic acids, which are inhibitory to microorganisms used in downstream processes.

For extraction and pre-treatment processes to be combined, the biomass post-extraction needs to be in a dry state to allow for downstream processing. With conventional organic solvents this would be challenging, as the biomass would need to be subjected to energy-intensive and time-consuming solvent removal processes to ensure that any residual solvent is removed. ScCO_2_ would be the ideal solvent in this scenario as, apart from it being non-toxic, cheap, and readily available, it leaves no solvent residue within the biomass post-extraction, meaning that the biomass can be directly passed on for processing [20].

This mini-review focusses on how scCO_2_ and high-pressure systems have been used for the valorisation of corn stover agricultural residues in extraction and pre-treatment. It also demonstrated how scCO_2_ can be used to obtain a variety of different chemicals including natural extracts, platform molecules, materials, and fuels from corn residues. Finally, the challenges and opportunities in the use of scCO_2_ and high-pressure technologies in a holistic biorefinery will be highlighted.

## Main text

### ScCO_2_ extraction of valuable compounds

ScCO_2_ is an efficient, fast, and most importantly clean technology for the extraction of natural products, which can add value to the biorefinery [21, 22]. Supercritical fluid extraction (SFE) technology is highly selective due to its solvent tuneability properties, has shorter extraction times, and could produce high purity products, while being benign to both human health and the environment [23–25]. Furthermore, SFE leaves no solvent residues, enabling the direct downstream processing of biomass without the need for solvent removal steps, which are both time-consuming as well as energy-intensive [23, 26]. Hence, this technology can be used as a good replacement to conventional organic solvent extractions, carried out with non-polar volatile organic compounds (VOCs), which apart from being environmentally harmful, can also degrade some of the extracted natural products [27, 28]. Residues of these solvents in the extract are also a concern, as the products are commonly used in food, pharmaceutical and cosmetic products [21, 28, 29].

Wax fractions from corn were traditionally obtained through the dipping of the seedlings and leaves in chloroform for 30 s [30–34]. Petroleum ether (30–60 °C boiling point) was also a solvent of choice; used in a number of studies with several extraction technologies including Soxhlet extractions, open-vessel microwave irradiation, and ultrasonic irradiation on powdered skin stalks of corn [35]. Previous work that employed scCO_2_ as the extraction medium focused more on the extraction of phytosterols, including ferulate-phytosterols from corn fibre oil and corn bran oil, a by-product of dry-milling of corn. Following optimization, the extractions were carried out at 34.5 MPa (345 bar) and 40 °C for 180 min [36, 37]. In both experiments, Taylor et al. carried out fractionation of the extract through supercritical fluid chromatography (SFC) using ethanol as a modifier, which was removed under reduced pressure at 60 °C [36, 37]. The use of polar modifiers such as ethanol can lead to changes in solvent polarity, which can result in greater extraction yields. However, the removal of such solvents for the product, is energy intensive and costly. Phytosterols have many crucial roles in several areas, including pharmaceuticals, for the production of therapeutic steroids, nutrition and cosmetics [38–41]. They are also thought to have a tremendous health benefit as they reduce serum cholesterol levels and in turn lower the risk of cardiovascular disease in humans [41].

**Fig. 2:**
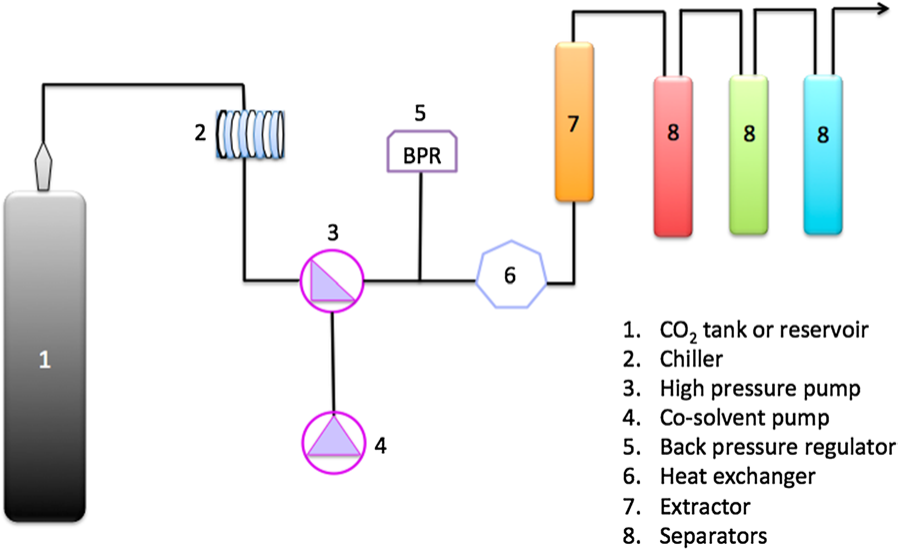
Simple schematic of an SFE apparatus, in which three separators were used in the fractionation step 400–150 bar, 50 °C (red Fraction A), 150–80 bar, 35 °C (green Fraction B), and at 80 bar-ATM and room temperature (blue Fraction C)

Attard et al. carried out a more in-depth study on the extraction of value-added products from corn stover as part of a holistic biorefinery [42]. The study investigated the hydrophobic constituents (waxes) of stover using scCO_2_ extraction as a green technology. Factorial experimental design (pressure range of 80–400 bars, a temperature range of 35–65 °C) showed optimal conditions of 400 bar and 65 °C with an extraction duration of 4 h. Supercritical extractions are frequently 1–4 h in duration, however typically most of the extractives had been removed from the biomass with the first 90 min. An increase in pressure leads to a greater extraction of waxes. This work demonstrated that the density of CO_2_ is a crucial parameter in the extraction process (0.33% at 80 bar/65 °C compared to 1.02% at 350 bar/50 °C). Although density has a significant effect on the extraction, there are other factors that influence the solubility of the hydrophobic molecules in CO_2_. The highest wax content (1.76%) was obtained at the density of 0.87 g cm^−1^ (400 bar/65 °C), the yield of which was significantly higher than those obtained at the greatest density of 0.9 g cm^−1^ (1.02%, 350 bar/50 °C). In this case, temperature was shown to also play an important role, as higher wax content was obtained at elevated temperatures (65 °C). Elevated temperatures facilitate melting of wax components, which can accelerate mass transfer and improve the extraction yield by promoting the solubility of the solute. This is dependent on the types of compounds being extracted and contrasts with several studies that demonstrate that greater temperature led to reduction in density and yield. Within this work the yields of lipids were consistent with other agricultural residues, but critically the extraction of such compounds would not be economically viable by themselves, the benefits imparted by supercritical extraction on the downstream processing of biomass residues must always be considered in any economic evaluation of such processes [42]. The study also illustrated the benefits of supercritical fractionation, which improves the extract selectivity leading to products of higher purity/quality. The first corn stover fraction, collected from the first separator, (fraction A in Fig. 2) was mainly composed of wax esters that have a great potential in lubricant formulations [43, 44]. The authors claimed that 42% of phytosterols were collected in Fraction B. (collected from the second separator); which is promising, since phytosterols have numerous health benefits and can be easily separated from the rest of the precipitated mixture [45]. The final fraction (collected from the third separator) was dominated by saturated and unsaturated fatty acids (atmospheric 50 °C). It is interesting to note that this fraction showed a great potential as a renewable defoamer in washing machine detergent formulations; which could make it a benign replacement to non-renewable anti-foaming agents, known to have a number of negative effects [46]. To achieve this on an industrial scale, greater yields are needed in shorter extraction times, which could be achieved using a higher flow rate of scCO_2_. This study was limited to 40 g of CO_2_/min, which is a low flow rate on an industrial scale, where there are systems capable of pumping 16 kg of CO_2_/min into 100 L separators, which would be equivalent to 80 g/minute in a 500 ml extractor. As such further work is needed to realise the potential of such extractions and applications at an industrial scale. Furthermore, other applications of saturated fatty acids are various and date to traditional applications, including soap manufacturing, as well as lubricating oil and oil additives while unsaturated fatty acids are well-known nutraceuticals [47–49].

ScCO_2_ is effective at extracting non-polar compounds, but the solvation power of the system can be enhanced through the addition of small amounts of polar co-solvents such as ethanol [29, 50, 51]. The addition of the co-solvent increases solvent power and enables the extraction more polar solutes from several kinds of biomass [29, 50, 51]. A drawback of adding a polar co-solvent to a non-polar scCO_2_ system is the reduction in selectivity [29]. Moreover, the addition of co-solvent requires further removal processes, but this could be aided by designing continuous feed and discharge systems for the solid matter, however, factors such as capital and operational costs must also be considered [29]. Further research is needed to investigate the effects of co-solvent addition on the profile of compounds extracted by scCO_2_ as well as the development of design and operation for using co-solvent.

To maximize the value of the biomass within a biorefinery context, extraction of valuable compounds should be performed prior to pre-treatment. Using scCO_2_ for extraction can remove the need for pre-treatment, thus saving time and cutting costs, furthermore this method does not produce potential inhibitors (to fermentation) as with conventional pre-treatment methods. Attard et al. demonstrated that scCO_2_ extraction of waxes from corn stover also increases ethanol production, following hydrolysis and fermentation of the stover, by 40% [42]. It was speculated that the removal of the epicuticular waxes increases the accessibility of lignocellulosic structure for enzymatic hydrolysis [42]. Furthermore, studies have shown that the epicuticular waxes of C_4_ biomass inhibit fermentation processes [52].

Liu et al. investigated the scCO_2_ extraction of flavonoids from the stigmata of corn flowers (*Maydis stigma*) [53]. Flavonoids from corn have been demonstrated to be highly efficient antioxidants and display significant physiological activities including resistance to attacks by insects. In addition, these extracts also exhibited anti-cancer and anti-diabetic properties. The effects of pressure, temperature and co-solvent quantity were studied using a combination of a Box-Behnken design coupled with response surface methodology (RSM). The flavonoid yield was increase by increasing the temperature at an early stage in the extraction, however, the reverse trend was observed when the temperature reached 50 °C under the range of pressure applied. Pressure plays an important role in the extraction; flavonoid yield increases when increasing the pressure from 25 to 45 MPa. It is noteworthy that the increasing of fluid density is probably the main mechanism leading to a higher flavonoid extract. To enhance the solubilities of polar compounds in CO_2_ by the addition of polar organic solvents, the extraction yield of flavonoids increases with greater proportions of co-solvents (20% aqueous ethanol) and reached a maximum when the quantity of co-solvent reached 2.5 mL/g. By computation analysis, the projected optimal conditions (2 h, 50.88 °C, 418 bar, and 20% aqueous ethanol) gave rise to a maximum predicted flavonoid content of 4.24 mg/g of dry *M. stigma*. The experimental yield from extracting five-times under the optimal conditions for 120 min with 0.4 mm particle size yielded 4.11 ± 0.38 mg/g of dry *M. stigma* and were shown to display high nitrite-scavenging ability, with the highest scavenging ability found to be 88% at a concentration of 500 µg/ml.

Monroy et al. conducted an optimization study, looking into the extraction of phenolic compounds from purple corn cobs [54]. Sequential extraction steps using scCO_2_, ethanol and water were conducted (in order of increasing polarity) using the same pressure and temperature conditions. The optimal conditions for extracting phenolics, in terms of pressure (259–541 bar) and temperature (36–64 °C), were conducted by Central Composite Rotatable Design (CCRD) while the process was optimized by means of RSM. The aqueous extract contained the highest yields and proportion of phenolics, while the ethanolic extracts contained the highest quantities of anthocyanins. The significant quantities of flavonoids, phenolics, anthocyanins gave rise to extracts with high antioxidant capacity, making them potential functional food ingredients.

Oruña-Concha and co-workers reported a comparison of scCO_2_ (350 bar, 60 °C, 1 h, 15% aqueous ethanol as co-solvent) and conventional extraction of carotenoids from sweet corn cobs (SCC) with methanol and repeatedly with hexane/acetone (1:1 v/v) [55]. ScCO_2_ showed significantly higher levels of carotenoids compared to conventional extraction. Moreover, scCO_2_ protected decomposition of carotenoids which are highly sensitive to light, air, heat, and pH by allowing the extraction without exposure to light and air, as the extraction is performed in an air-tight and closed chamber. Three carotenoids were identified in the extract consisting of lutein, zeaxanthin, and β-carotene. The content of zeaxanthin and lutein in SCC was twice in scCO_2_ extraction, while the content of β-carotene was three times higher in scCO_2_ extraction, as compared to conventional extraction. In addition, supercritical fluid can enhance the penetration into porous solid material, resulting in a faster effective extraction process by the combination of high diffusivity and low viscosity.

The survey of the above works related to the effect of scCO_2_extraction pressure on the global extract yield demonstrated an increase with greater pressure. In addition, it was observed that the solubility increased with pressure above 300 bar. The increase of temperature generally resulted the decrease of the solvents density thus reducing the solvent power of the supercritical solvent, leading to the decrease of the extraction yield, however, in the case of some waxes the temperature played a positive effect on the extraction yield [42]. Moreover, the increase of temperature enhances the vapour pressure of the compounds to pass in the fluid phase and increasing them to be extracted [29]. scCO_2_ technology can prevent the decomposition of thermally labile chemicals [55]. The addition of small amounts of polar co-solvents such as water and ethanol enhanced the extraction of polar compounds such as carotenoids and phenolic compounds from corn stover [53–55]. However, the addition of such co-solvents could reduce the selectivity and negates the advantage of solventless operation [29].

Economic studies on the scCO_2_ extraction of waxes from corn stover as part of an integrated biorefinery have been conducted and it has been shown that extraction of waxes from corn stover could be cost effective [42, 56]. The economic study considered the fixed capital investment (cost of the industrial supercritical unit), operational labour costs, the cost of the raw material (the cost of the corn stover which includes harvesting, storage, transportation costs amongst others), utility costs (energy and electricity required for the extraction process) and waste costs. The lowest cost of manufacture (COM) was estimated to be €4.56 per kg of corn stover wax (whereby the corn stover biomass is subjected to combustion following extraction) [56]. This cost could be further reduced if other added value compounds are obtained from downstream processes, such as fermentation, prior to combustion. Wax reports from 2018 show that the average market price of common non-petroleum derived waxes was estimated to be €4.72/kg of wax. Candelila wax, carnauba wax and beeswax were found to be €3.97 per kg of wax, €7.85 per kg of wax and €8.99 per kg of wax respectively [57]. This indicates that the COM for the corn stover wax can be economically viable. Furthermore, there is a variation in the market price of wax depending on its purity and use. Since scCO_2_ extraction is a more selective technique in comparison to traditional organic solvent extraction (several additional compounds are extracted along with the wax including pigments such as chlorophyll), scCO_2_-extracted waxes are of higher purity or quality and would therefore have a higher market price. Hence, scCO_2_ could be an effective step in the extraction of these waxes within a green biorefinery, prior to destructive downstream processing steps, such as microwave technology.

Considering the recent developments in the scCO_2_ pre-treatments of corn residues, these processes demonstrate several advantages for extraction: (i) it is easily available, non-toxic, and easily recycled; (ii) extraction conditions are typically mild, requiring moderate temperatures (< 100 °C); (iii) the separation of solvent and product is easily performed by a simple release of pressure; (iv) avoids the use of potentially hazardous and environmentally damaging organic solvents; (v) such processes can incorporate fractional separation to improve extraction selectivity and generates products of higher value; (vi) scCO_2_ extraction enhances the downstream processing of the corn stover biomass and (vii) scCO_2_ extraction is techno-economically feasible. Moreover, scCO_2_ extraction can also be applied as a pre-treatment technique to enhance enzyme accessibility to cellulose biomass.

Supercritical fluids can be applied for corn stover valorisation as a reactive extraction method to afford value-added compounds such as steroids, waxes, phenolic compounds, and pigments with their application in pharmaceutical or food products. Some promising outcomes of using supercritical fluid extraction are listed in Table 1 that summarizes the recovery results of various natural products from corn stover.

**Table 1:** Extraction conditions and outcomes for the supercritical fluid pre-treatment of corn stover

Types of biomass	Extraction condition	Outcomes of extracted corn stover	Year	Refs
Stigmata of corn flower	50.88 °C, 41.8 MPa, 2 h, flow rate 20 L/h, 20% aq. ethanol as co-solvent, 2.488 mL/g	Flavonoids	2011	[53]
Corn	55 °C, 45 MPa, 2 h, flow rate 8000 mL/min	β-sitosterol, stigmasterol, and campesterol	2015	[39]
Corn stover	65 °C, 400 bars, 4 h, flow rate 300 g/min, 3 fractionations, 150 bar/50 °C, 85 bar/35 °C, and ATM/50 °C	Hydrocarbons, sat. fatty acids, unsat. fatty acids, fatty alcohols, sterols, and steroid ketones	2015	[42]
Purple corn cob	45 °C, 420 bar, flow rate 1.65 g/min Ethanol as co-solvent, flow rate 0.395 g/min, 50 °C, 400 bar, flow rate 1.65 g/min (for Anthocyanins) Water as co-solvent, flow rate 0.5 mL/min 65 °C, 450 bar, flow rate 1.65 g/min (for Flavonoids) Water as co-solvent, flow rate 0.5 mL/min (for phenolics)	Anthocyanins, flavonoids and phenolics	2016	[54]
Sweet corn cob	60 °C, 350 bar, 1 h, flow rate 15 g/min, 15% aq. ethanol as co-solvent	Lutein, zeaxanthin, and β-carotene	2019	[55]

### CO_2_-H_2_O pre-treatment of corn stover

The combination of high-pressure CO_2_ with water has been researched extensively in acid-catalysed reactions. This is now regarded as an important pre-treatment technology in biomass valorisation [5]. The inherent moisture content of biomass combined with CO_2_ at appropriate conditions (pressure and temperature) can lead to enhanced sugar yields [5]. The presence of water forms carbonic acid (Eq. 1), which may hydrolyse hemicellulose and break the hydrogen bonding between cellulose, hemicellulose, and lignin [58].

$$ {\text{CO}}_{{2}} {\text{ + 2H}}_{{2}} {\text{O }} \leftrightarrow {\text{ HCO}}_{{3}}^{ - } {\text{ + H}}_{{3}} {\text{O}}^{ + } $$

$$ {\text{HCO}}_{{3}}^{ - } {\text{ + H}}_{{2}} {\text{O }} \leftrightarrow {\text{ CO}}_{{3}}^{{2 - }} {\text{ + H}}_{{3}} {\text{O}}^{ + } $$

The unstable carbonic acid easily dissociates to a hydronium ion. The high concentration of hydronium ions causes the pH of the system to decrease (slightly above 3); resulting in the dissolution and hydrolysis of hemicellulose to C_5_ sugars [59–61]. Furthermore, a decrease in the pH also leads to an increase in the enzyme digestibility of cellulose [62]. Therefore, CO_2_-H_2_O technology demonstrates the same advantages as conventional acid catalysis of biomass without the need for a neutralization step following the reaction [63]. Van Walsum and co-workers demonstrated that carbonic acid could enhance the hydrolysis of hemicellulose fractions in corn stover at elevated temperatures (above 200 °C) compared to water-only pre-treatment [64]. This was also was consistent with reported work on carbonic acid pre-treatment of beech wood [62]. However, carbonic acid showed no enhancement on hydrolysis of aspen wood [65]. Although carbonic acid could be a viable reagent for promising hydrolysis of lignocellulosic biomass, the application of high-pressure CO_2_-H_2_O technology shows more benefits. Importantly, carbonic acid is prepared in situ and reduces costs and risks in transportation of acids. Besides, the presence of carbonic acid showed a higher final pH than the samples prepared with water alone [64, 65]. Moreover, the utilisation of CO_2_–H_2_O binary system is the *in-situ* formation that does not face an environmental issue due to CO_2_ is no longer present in the reaction system after the depressurisation [5, 61] and pre-treatment can aid the enzymatic process without the necessity for pH adjustment of the medium for the hydrolysis [5].

Van Walsum et al. published an investigation on dissolving carbon dioxide in liquid hot water that yielded carbonic acid to enhance the hydrolysis of biomass including corn stover [64, 66, 67]. Morais et al. published a review on carbon dioxide pre-treatment processes of various biomass in the presence of water which focused on the role of high-pressure carbon dioxide as a tool to enhance the enzymatic digestibility to produce biofuels and biochemicals from lignocellulosic or starch biomass [5]. The present mini-review presents the current stage of utilization of high-pressure carbon dioxide with water to improve enzymatic hydrolysis of lignocellulosic biomass which focus on corn stover. Table 2 demonstrates the pre-treatment with CO_2_ can increase the efficiency of hydrolysis that indicates the use of high-pressure CO_2_ as a solvent that can be used in a great variety of applications within the biorefinery concept for pre-treatment of corn stover.

**Table 2:** Pre-treatment conditions and outcomes for the supercritical fluid pre-treatment of corn stover

Types of biomass	Pre-treatment condition	Outcomes of pre-treated corn stover	Year	Refs
Corn stover	160–200 °C, 13–17 MPa, 40–80 min, water:ethanol as co-solvent	Removal of lignin reached to 90.05% and increased the enzyme accessibility	2013	[69]
Corn stover	120 °C, 1450 psi, 30 min, ethanol as co-solvent	Increased sugar yield of 2 mm and 4 mm particles at a higher pressure, but decreased sugar yield of 1 mm as pressure increased	2018	[70]
Corn stover, corn cob, and sorghum stalk (75% moisture)	50–80 °C, 17.5–25 MPa, 12–60 h	Pre-treatment significantly reduced the temperature from 160–170 to 50–80 °C and increased the enzyme accessibility 3- or fourfold compared to unpretreated	2019	[68]
Corn straw (10.7% moisture)	170 °C, 5 MPa, 40 min	Enhanced enzyme hydrolysis with little lignin degradation	2019	[71]

Yin et al*.* reported CO_2_ pre-treatment of various agricultural crop residues including corn stover, corn cob, and sorghum stalk with a moisture content of 75% by weight [68]. Compared to the previous study, the pre-treatment temperature can be decreased from high (160–170 °C) to low (50–80 °C) temperatures by extending the pre-treatment time from 0.5–2 h to 24–48 h. The moisture content is a very important factor in CO_2_ pre-treatment, where wetting, softening, and swelling of the lignocellulose enhances the CO_2_ penetration into the lignocellulose, thereby increase the surface area of the biomass making it accessible to enzymatic hydrolysis, which can lead to higher sugar yields of three to four-fold compared to those of the raw materials.

CO_2_ with a modifier system made up of water–ethanol was used by Zhang et al*.* as a form of pre-treatment for corn stover [69]. They demonstrated the highly efficient process of delignification in CO_2_ with ethanol–water (2:1 v:v) as co-solvent during pre-treatment of corn stover. The pre-treatment temperature was the most significant factor for lignin removal which can remove > 80% of lignin. The highest glucose yield of 80.5% was obtained at optimal pre-treatment conditions (180 °C, 60 min); however, it can be increased to 92% through the removal of lignin residues deposited on the fibre surface by washing with an ethanol–water solution.

Apart from the combination of CO_2_ and H_2_O, Yang et al*.* studied CO_2_ pre-treatment of corn stover for biofuel production using ethanol as a co-solvent [70]. They reported the effect of particle size on CO_2_ pre-treatment, with three particle sizes (1, 2 and 4 mm). Higher yields were obtained with smaller particles sizes, with the highest sugar yield (0.115 g glucose per 1 g of dry biomass) obtained at 100 bar, 120 °C for 30 min for the 1 mm particle size. This is 16.62% and 10.39% higher than the 4 mm and 2 mm corn stover biomass particle sizes, respectively. Thus, particle size range (narrow and wide) needs to be taken into consideration in pre-treatment of cellulosic biomass for biofuel production.

In another recent example, Wu et al*.* reported subcritical CO_2_-assisted autohydrolysis pre-treatment for oligosaccharides and fermentable sugars production from corn straw (water content was 10.7%) [71]. Xylose was the main sugar in the prehydrolysate and the largest yield of about 90.2% of xylo-oligosaccharides (XOS) was obtained; furthermore, the functional XOS (DP < 5) was about 40% of the total XOS. Few inhibitors were generated during the pre-treatment but there was almost no effect on subsequent fermentation. In addition, the maximum glucose yield in enzymatic hydrolysis was 32.8 g/100 g corn straw (90.6%) and mostly lignin (21.2 g/100 g corn straw, 89.4%) was recovered in the enzymatic hydrolysis residue. As such, CO_2_-assisted autohydrolysis pre-treatment was an effective method to enhance corn straw enzymatic hydrolysis with little lignin degradation.

It is important to emphasise that all the studies presented above were performed the utilisation of high-pressure carbon dioxide with a co-solvent system such as water/ethanol for pre-treatment of corn stover and importantly demonstrates a vital role in increasing the yields of hydrolysis. The addition of co-solvents in scCO_2_ technology is a promising alternative to alter solvent properties, thus modifying solubility and polarity factors. The high-pressure CO_2_ enhances lignin cleavage and promotes the capacity of co-solvent to dissolve lignin. Therefore, the effects of high-pressure CO_2_ enhance the delignification of biomass and reduce mass resistance that enhance substrate accessibility to the hydrolysis process. A decrease in particle size can create more surface area and shorten the diffusion paths in a solid matrix of biomass that enhances the performance of CO_2_ pre-treatment. The use of water can break down the bonds between lignin and hemicellulose scaffolds and affects changes in the biomass structure such as wetting, softening, and swelling that enhance the accessibility of CO_2_ to the solid matrix which results in hydrolysis improvement. One of the economic advantages of the utilisation of scCO_2_ for pre-treatment is that no or few fermentation inhibitors are produced leading the biomass ready for hydrolysis without the need for any separation or purification processes.

Supercritical fluid technologies have been used for pre-treatment of different lignocellulosic biomass, including corn stover. Pre-treatment of lignocellulosic materials with scCO_2_ show no considerable change in the biomass structure, compared with the unpretreated material [72]. However, the combination of the CO_2_ technique and moisture pre-treatment results in significant changes in the biomass structure. Moreover, the presence of moisture does not affect the CO_2_ to reach a supercritical state but can transform scCO_2_ to a carbonic acid which acts as a catalyst and increase the enzymatic hydrolysis rate of the biomass.

### Challenges for the application of supercritical fluids in holistic biorefinery

Cost assessments and feasibility studies need to be further conducted as cost is an important barrier to overcome for widespread use. Green technologies such as scCO_2_ extraction or pre-treatment are still perceived to be expensive, and it is important that this preconception is overcome. There have been studies which have focused on the economic viability of supercritical extraction as a green alternative to extract essential oils and waxes (including stover) from a variety of biomass [56]. However, few economic assessments have been published on the industrial scale use of scCO_2_ extractions. As such, the financial evaluations of large-scale supercritical carbon dioxide processes for corn residues are warranted and essential to further demonstrate their economic viability at scale. In fact, the demonstration of supercritical extraction or pre-treatment at scale still has not been achieved for corn residues and is another important area that needs to be further investigated. Importantly, there are currently new scCO_2_ extraction systems being developed at cheaper costs with higher flow rates of throughput, so the future economics of these processes is looking promising.

Recently, a procedure for the techno-economic analysis of supercritical fluid processes has been reported [73]. This report presents a critical analysis and a review of several techno-economic assessments applied for fuel production which relates to supercritical fluid conditions of CO_2_, water, and alcohol. The integration of mass and energy in a biorefinery concept can be designed to reduce the estimated capital cost of the processes. Moreover, the use of co-solvents or suitable catalysts combined with scCO_2_ would result in lower temperatures and pressures, thus potentially resulting in lower energy consumption and capital costs.

Commercially, supercritical technologies have been successfully employed for the industrial extraction of hops, coffee and, more recently, in the extraction of cannabinoids from cannabis [26]. Importantly, the costs associated with supercritical extraction are mitigated by the high value of the products from hops, coffee, and cannabis. In addition, the selectivity of the supercritical carbon dioxide extraction is beneficial for production of a premium product, as no pigments and other impurities are co-extracted. In contrast, many products from the extraction of agricultural residues are still yet to find high value applications, potentially limiting the economic appeal of supercritical extraction. Therefore, it is key to not look at supercritical extraction/pre-treatment of agricultural residues as a standalone technology but as an integrated tool within a biorefinery, thus providing additional benefits of better biomass downstream processing.

## Conclusions

Supercritical fluid extraction and high-pressure pre-treatment are important technologies for integration into holistic corn stover biorefineries. These technologies can aid in the valorisation of lignocellulosic resources to produce a variety of different chemicals included value-added chemicals, platform molecules, materials, and fuels. The use of high pressures carbon dioxide technologies such as liquid and scCO_2_ are an economic challenge for utilization at a large scale, but the benefits may justify its implementation, especially when applied together with other technological approaches that would help to the development of holistic processes for valorisation of all available agricultural residues. Future developments in the design of supercritical systems may add in reducing capital and operational costs.

## Data Availability

Not applicable.

## Abbreviations

CCRD: Central composite rotatable design
COM: Cost of manufacture
DP: Degree of polymerization
RSM: Response surface methodology
SCC: Sweet corn cob
scCO_2_: Supercritical carbon dioxide
SFC: Supercritical fluid chromatography
SFE: Supercritical fluid extraction
VOCs: Volatile organic compounds
XOS: Xylo-oligosaccharides
